# Cost-Effective Respiratory Virus Testing

**DOI:** 10.1128/JCM.00373-19

**Published:** 2019-08-26

**Authors:** B. A. Pinsky, R. T. Hayden

**Affiliations:** a Department of Pathology, Stanford University School of Medicine, Stanford, California, USA; b Department of Medicine, Division of Infectious Diseases and Geographic Medicine, Stanford University School of Medicine, Stanford, California, USA; c Department of Pathology, St. Jude Children’s Research Hospital, Memphis, Tennessee, USA; Emory University

**Keywords:** respiratory viruses

## Abstract

The timely and accurate diagnosis of respiratory virus infections has the potential to optimize downstream (posttesting) use of limited health care resources, including antibiotics, antivirals, ancillary testing, and inpatient and emergency department beds. Cost-effective algorithms for respiratory virus testing must take into consideration numerous factors, including which patients should be tested, what testing should be performed (for example, antigen testing versus reverse transcription-PCR testing or influenza A/B testing versus testing with a comprehensive respiratory virus panel), and the turnaround time necessary to achieve the desired posttesting outcomes. Despite the clinical impact of respiratory virus infections, the cost-effectiveness of respiratory virus testing is incompletely understood. In this article, we review the literature pertaining to the cost-effectiveness of respiratory virus testing in pediatric and adult patient populations, in emergency department, outpatient, and inpatient clinical settings. Furthermore, we consider the cost-effectiveness of a variety of testing methods, including rapid antigen tests, direct fluorescent antibody assays, and nucleic acid amplification tests.

## Abstract (precis)

The timely and accurate diagnosis of respiratory virus infections has the potential to optimize downstream (posttesting) use of limited health care resources, including antibiotics, antivirals, ancillary testing, and inpatient and emergency department beds.

## GOAL

The goal of cost-effective respiratory virus testing is to ensure patient health while optimizing the use of limited health care resources.

## DECISION TO TEST

The first decision point encountered in the quest for cost-effective respiratory virus testing is the determination of whether a patient requires testing. This determination involves a clinical interpretation that considers presenting signs and symptoms, the day of illness at presentation (given the diminished efficacy of anti-influenza therapies after 48 h), and risk factors, such as the extremes of age or immunocompromise, that may predispose patients to severe respiratory disease. The U.S. Centers for Disease Control and Prevention (CDC) encapsulate this process for influenza virus testing in a decision tree that includes clinical presentation, hospital admission, and whether the testing results will influence clinical management (https://www.cdc.gov/flu/professionals/diagnosis/consider-influenza-testing.htm).

Note that the clinical signs and symptoms that define influenza-like illness (ILI) are neither sensitive (sensitivity of ∼60%) nor specific (specificity of 0 to 90%) (1). Furthermore, these ILI definitions differ slightly based on which agency or research group sets the case definition (https://www.cdc.gov/vaccines/pubs/surv-manual/chpt06-influenza.html) (1). For example, the U.S. CDC define ILI as fever of ≥100°F (37.8°C) and cough and/or sore throat, whereas the World Health Organization (WHO) defines ILI as an acute respiratory illness with a measured temperature of ≥38°C and cough, with onset within the past 10 days. How ILI is defined affects influenza surveillance (2) and oseltamivir use (3) and therefore may also effect decision-tree-based models for cost-effective respiratory virus testing.

Nevertheless, cost-benefit modeling suggests that an approach of testing and then treating, compared with no testing/empirical therapy, is the most cost-effective strategy for moderate influenza prevalence (4, 5) or low influenza prevalence combined with a low-to-moderate risk of hospitalization (6). Another cost-benefit model demonstrated that using reverse transcription (RT)-PCR results to guide antiviral therapy in older adults (≥65 years of age) was the most cost-effective strategy when influenza prevalence was moderate to high (7). These models predict that the cost-effectiveness of influenza testing varies significantly based on disease prevalence, highlighting the importance of epidemiological monitoring to optimize test utilization. Limitations of the modeling approach include the use of parameters that may not represent real-world clinical behavior, such as assuming that testing does not affect hospital admission or omitting certain considerations of cumulative costs, such as the cost of unnecessary testing in a missed diagnosis of influenza. However, extending these models to account for additional respiratory viruses will likely further refine our understanding of the variables that affect the cost-effectiveness of respiratory virus testing and may allow us to provide more sophisticated decision trees for cost-effective clinical management. Practical recommendations for cost-effective testing include testing only once per episode, unless signs and/or symptoms change, and eliminating repeat testing to confirm coinfections.

## SPECIMEN SELECTION

Once a decision has been made to test, the appropriate respiratory tract specimen must be collected (reviewed in detail in reference 8). In order to maximize detection of respiratory viruses in the upper respiratory tract, sampling of the posterior nasopharynx via nylon flocked swab, wash, or aspirate is recommended. Although a number of studies have demonstrated that nasopharyngeal aspirates are more sensitive than specimens collected with flocked swabs, other studies showed that these collection methods result in similar diagnostic performance (9–12). Nasal swabs generally result in lower overall sensitivity, compared to collection methods that sample the nasopharynx; however, performance may vary based on the virus evaluated, the patient population tested, and the method used for detection (13). If an FDA-cleared respiratory virus detection assay is used, then the manufacturer’s instructions for collection, transport, and processing should be verified and followed. Lower respiratory tract specimens, such as bronchoalveolar lavage fluid samples, are frequently validated by laboratories, particularly for immunocompromised patients. A syndromic pneumonia panel (BioFire FilmArray), including both viruses and bacteria, has been FDA cleared for lower respiratory tract specimens. Nonrespiratory specimen types are not recommended for routine testing.

## TESTING METHODS

Once the specimen type has been decided, the type of respiratory virus test to perform must also be considered. Methods for clinical testing of respiratory viruses include primarily rapid antigen tests and nucleic acid amplification tests (NAATs), although some laboratories continue to perform direct fluorescent antibody (DFA) testing and viral culture (14). The technical details of these methods are described elsewhere (15, 16). Reagent and instrument costs, as well as labor costs to perform the testing, are important components of the cost-effectiveness analysis. Reagent costs are dependent on the test volume and, for tests that require instrumentation, reagent costs may differ if the capital equipment is purchased or obtained via reagent rental. In addition, labor markets differ markedly throughout the United States and globally, and high reagent costs may be justified in some high-cost labor markets if the need for staffing is reduced.

A list of FDA-cleared rapid antigen tests is provided in Table 1 (see https://www.cdc.gov/flu/professionals/diagnosis/table-ridt.html for the most up-to-date information), which includes both waived and nonwaived rapid antigen tests. As defined by the Clinical Laboratory Improvement Amendments (CLIA), waived tests are categorized as “simple laboratory examinations and procedures that have an insignificant risk of an erroneous result” (94). From a practical perspective, tests with a waiver from the FDA can be performed at outpatient clinics and other facilities that have obtained a CLIA certificate of waiver. These sites are not subject to the same regulatory requirements as laboratories that perform moderate- and high-complexity testing.

**TABLE 1:** FDA-cleared influenza A/B rapid antigen tests

Manufacturer	Product	Platform/instrument	Approved specimen type(s)^*a*^
Abbott	Binax Now Influenza A & B Card 2	Alere reader	**NPS, NS direct**
Becton Dickinson & Co.	BD Veritor Flu A + B	BD Veritor reader, BD Veritor Plus analyzer	**NPS, NS direct,** NPW, NA, NPS in VTM
Quidel Corp.	Sofia Influenza A + B FIA	Sofia FIA analyzer, Sofia 2 FIA analyzer	**NS, NPS, NPA, NPW direct, NPW in VTM**
Quidel Corp.	QuickVue Influenza A + B	NA	**NPS, NS direct**
Princeton BioMeditech Corp.	BioSign Flu A & B, Labsco Advantage Flu A & B, LifeSign Status Flu A & B, OraSure QuickFlu Rapid A + B, Polymedco Poly stat Flu A&B, Sekisui Diagnostics OSOM Ultra Flu A & B, Meridian BioScience ImmunoCard Stat! Flu A&B, McKesson Consult Diagnostics Influenza A&B	NA	**NS, NPS direct,** NPA, NPW
Remel/Thermo Fisher	Xpect Flu A & B	NA	Nasal wash

a NPS, nasopharyngeal swab; NS, nasal swab; NA, nasal aspirate; NPA, nasopharyngeal aspirate; NPW, nasopharyngeal wash; VTM, viral transport medium; NA, not applicable. Specimen types listed in bold are CLIA waived.

Table 2 includes FDA-cleared, CLIA-waived, sample-to-answer, respiratory virus NAATs, whereas Table 3 includes selected FDA-cleared, nonwaived, respiratory virus NAATs. For additional details regarding these tests, as well as other FDA-cleared reagents for the molecular detection of respiratory viruses, see the microbial tests tab at https://www.fda.gov/MedicalDevices/ProductsandMedicalProcedures/InVitroDiagnostics/ucm330711.htm.

**TABLE 2:** FDA-cleared and CLIA-waived respiratory virus NAATs

Manufacturer	Product	Platform/instrument	Approved specimen type(s)^*a*^
Abbott	Alere i Influenza A&B 2	Alere i (ID NOW)	NS direct, NPS direct, NS, NPS
Abbott	Alere i Influenza RSV	Alere i (ID NOW)	NPS direct, NPS
BioFire Diagnostics	BioFire FilmArray respiratory panel EZ	BioFire FilmArray	NPS
Cepheid	Xpert Xpress Flu	GeneXpert Xpress	NS, NPS
Cepheid	Xpert Xpress Flu/RSV	GeneXpert Xpress	NS, NPS
Mesa Biotech, Inc.	Accula Flu A/Flu B	Accula Dock	NS
Mesa Biotech, Inc.	Accula RSV	Accula Dock	NS
Roche	cobas Liat Influenza A/B assay	cobas Liat	NPS
Roche	cobas Liat Influenza A/B & RSV assay	cobas Liat	NPS
Sekisui Diagnostics	Silaris Influenza A&B test	Silaris Dock	NS direct

a NPS, nasopharyngeal swab; NS, nasal swab.

**TABLE 3:** Selected FDA-cleared but nonwaived respiratory virus NAATs

Manufacturer	Product	Platform/instrument	Approved specimen type(s)^*a*^
BioFire Diagnostics	BioFire FilmArray respiratory panel 2	BioFire FilmArray	NPS
BioFire Diagnostics	BioFire FilmArray respiratory panel	BioFire FilmArray	NPS
BioFire Diagnostics	BioFire FilmArray pneumonia panel	BioFire FilmArray	IS, TRA, BAL fluid
Cepheid	Xpert Flu/RSV XC	GeneXpert	NPS, NPA, NPW
Cepheid	Xpert Xpress Flu	GeneXpert	NS, NPS
Cepheid	Xpert Xpress Flu/RSV	GeneXpert	NS, NPS
Diasorin	Simplexa Flu A/B & RSV Direct	Liason MDX	NPS
GenMark Diagnostics	ePlex respiratory pathogen panel	ePlex	NPS
Hologic, Inc.	Panther Fusion Flu A/B/RSV assay	Panther Fusion	NPS
Hologic, Inc.	Panther Fusion AdV/HMPV/RV assay	Panther Fusion	NPS
Hologic, Inc.	Panther Fusion Paraflu assay	Panther Fusion	NPS
Luminex	Aries Flu A/B & RSV assay	Aries	NPS
Luminex	Verigene Respiratory Pathogens Flex	Verigene reader and processor SP	NPS
Qiagen	QIAstat-Dx respiratory panel	QIAstat-Dx	NPS
Quidel	Solana Influenza A + B assay	Solana	NS, NPS
Quidel	Solana Influenza RSV+hMPV assay	Solana	NS, NPS

a NPS, nasopharyngeal swab; NS, nasal swab; NPA, nasopharyngeal aspirate; NPW, nasopharyngeal wash; IS, induced/expectorated sputum; TRA, tracheal aspirate; BAL, bronchoalveolar lavage.

It is important to note that critical components of cost-benefit analyses are the performance characteristics of the test being evaluated, as this information allows estimation of the impact of false-positive and false-negative results. For example, a rapid antigen test with lower sensitivity and similar specificity, compared to a NAAT, may be less cost-effective despite lower costs for reagents, equipment, and labor.

As has been well described in the literature, rapid antigen tests for influenza demonstrate poor to moderate sensitivity, depending on the particular assay and the circulating strain. A meta-analysis of influenza rapid antigen tests revealed pooled sensitivities of 64.6% for influenza A (95% confidence interval [CI], 59.0% to 70.1%) and 52.2% for influenza B (95% CI, 45.0% to 59.3%), with a combined pooled specificity of 98.2% (95% CI, 97.5% to 98.7%) (17). That analysis was completed prior to the introduction of next-generation digital antigen immunoassays with automated detection, such as the Quidel Sofia and BD Veritor systems, which generally show improved sensitivity, compared to conventional lateral flow rapid immunoassays that rely on visual detection by human readers (15, 18). A subsequent meta-analysis reported digital antigen immunoassay pooled sensitivities of 80.0% (95% credible interval [CrI], 73.4% to 85.6%) for influenza A and 76.8% (95% CrI, 65.4% to 85.4%) for influenza B, with a combined pooled specificity of >98% (19). Using the same methodology, those authors described pooled sensitivities for conventional rapid antigen immunoassays of 54.4% (95% CrI, 48.9% to 59.8%) for influenza A and 53.2% (95% CrI, 41.7% to 64.4%) for influenza B, with similarly high pooled specificity. An additional meta-analysis also observed higher pooled sensitivity for digital antigen immunoassays than for conventional rapid influenza antigen tests (20). However, the CDC do not yet distinguish between these antigen detection methods and recommend that patients who present with a syndrome consistent with influenza and have a negative rapid antigen test result should either receive a confirmatory RT-PCR test or be treated as if they have influenza, due to the overall limited sensitivity of antigen testing. Influenza rapid antigen tests were recently reclassified by the FDA in order to meet minimum performance standards, and compliance was mandatory by 12 January 2018 (21).

A meta-analysis of respiratory syncytial virus (RSV) rapid antigen tests that included assays with automated readers revealed a pooled sensitivity of 80.0% (95% CI, 76.0% to 83.0%) and a pooled specificity of 97.0% (95% CI, 96.0% to 98.0%) (22). Interestingly, the American Academy of Pediatrics does not recommend testing for RSV or other respiratory viruses in children with a clinical diagnosis of bronchiolitis (23), as the authors assert that, at an individual patient level, the value of identifying a specific viral etiology has not been demonstrated. However, monitoring the start and end of the RSV season is acknowledged to be important for the optimal administration of RSV passive immunization (palivizumab) among high-risk pediatric patients (24).

As the field of clinical virology has transformed into clinical molecular virology, NAATs have become the reference method for the diagnosis of respiratory virus infections, generally demonstrating superior sensitivity without a loss of specificity, compared to rapid antigen testing (16, 18, 25). With the development of numerous sample-to-answer systems, including the Cepheid GeneXpert, BioFire FilmArray, and GenMark ePlex systems, as well as FDA approval of waived respiratory virus NAATs such as the Alere i (26–35), cobas Liat (26, 34, 36–43), and Xpert Xpress (35, 39, 41–48) tests, laboratories and facilities using point-of-care testing no longer need to compromise performance for simplicity, ease of use, and rapid test turnaround (49, 50). A meta-analysis revealed that rapid NAATs have pooled sensitivities of 91.6% (95% CrI, 84.9% to 95.9%) for influenza A and 95.4% (95% CrI, 87.3% to 98.7%) for influenza B, with pooled specificities of >99% (19). There remain, however, further considerations in developing algorithms for cost-effective respiratory virus testing, including the number of targets required to be included in the panel and the rapidity with which results must be reported.

**FIG 1:**
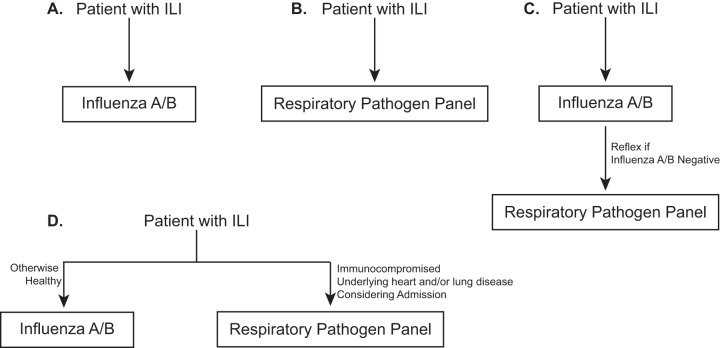
Respiratory virus testing algorithms. (A) All patients with ILI are tested using an influenza A/B test. (B) All patients with ILI are tested with a respiratory pathogen panel. (C) Patients with ILI are tested with an influenza A/B test and, if the results are negative, then reflex testing with a respiratory pathogen panel is performed. (D) Patients with ILI are tested with an influenza A/B test or a respiratory pathogen panel depending on underlying diseases and the severity of the presentation.

Determining the optimal panel for the diagnosis of respiratory virus infections continues to be an area of active discussion (51–54). Options include primary testing for influenza A/B, primary syndromic respiratory panel testing, or some combination (for example, influenza A/B testing with reflex testing with a respiratory virus panel if influenza results are negative) (Fig. 1). Variables that may be considered include patient age, immune status, location (inpatient versus outpatient), acuity of infection, influenza vaccination status, and virus prevalence/seasonality. For inpatients, infection control and prevention factors must also be considered, including decisions about isolation and cohorting.

Data regarding the utility and cost-effectiveness of these potential algorithmic approaches are relatively limited, although a recent observational study of adult outpatients at a large Veterans Affairs (VA) medical center suggested that testing for influenza viruses alone may be more cost-effective than multiplex respiratory pathogen testing in this patient population (55). In contrast, a retrospective, case-control study of pediatric inpatients revealed that multiplex testing with the BioFire FilmArray system was associated with reduced antibiotic use and decreased chest radiographs (56). However, a prospective assessment of multiplex respiratory panel testing for hospitalized adults revealed that the diagnosis of influenza virus infection was associated with reduced duration of hospitalization and appropriate antiviral management but detection of other respiratory viruses was not significantly associated with study outcome measures (57). Another study, using a decision-analysis approach in a pediatric patient population, concluded that testing using the Luminex xTag respiratory virus panel NAAT was less costly than other testing strategies when the prevalence of infection was ≥11%, with savings being primarily due to a reduction in the duration of hospitalization (58). Other groups have demonstrated potential laboratory cost savings associated with syndromic respiratory virus testing (59, 60) or a two-stage algorithm involving Quidel Sofia influenza antigen testing with reflex testing of negative samples with the BioFire FilmArray system (61). The contributions of the myriad coinfections diagnosed by syndromic testing to cost-effective respiratory virus testing remain an area of active investigation.

## COST-EFFECTIVENESS OF RAPID TESTING

The final variable to consider for cost-effective respiratory virus testing is turnaround time, which has been studied primarily in the context of rapid antigen tests, although data are beginning to be collected using NAATs, with both targeted testing and large respiratory virus panels. Given the large number of studies on this topic, this section has been divided into subsections describing observational studies and randomized controlled trials (RCTs), with further organization based on the clinical setting (emergency department [ED]/outpatient versus inpatient testing).

### Observational studies.

**Emergency departments and outpatient clinics.** In EDs and outpatient clinics, the clinical utility and effectiveness of rapid respiratory virus testing have been studied in several observational studies. Patients in these settings with positive rapid influenza antigen immunoassay results have been shown to receive fewer antibiotics, to undergo fewer diagnostic tests, to be more likely to receive antiviral therapy, and to be less likely to be hospitalized than patients whose rapid test results are negative. One or more of these outcomes have been demonstrated in both pediatric (62, 63) and adult (64) patient populations. Similar findings were observed when positive influenza results were reported before rather than after ED discharge (65) and when positive influenza results were available before rather than after the ED physician’s examination (66). Furthermore, for both adult patients with specimens submitted within 48 h after presentation (67) and pediatric patients admitted from the ED (68), the rapid diagnosis of influenza using the FilmArray respiratory virus panel was associated with decreased length of stay and duration of antimicrobial use. An economic modeling analysis of rapid influenza diagnosis using the FilmArray system in a pediatric ED setting indicated that rapid NAAT analysis for influenza was the most cost-effective strategy, compared to conventional influenza NAAT analysis, DFA testing, and rapid antigen testing (69). In addition, a prospective study evaluating the impact of rapid cobas Liat influenza A/B testing on physician decision-making in a mixed adult/pediatric ED and an adult ED suggested the potential for significant cost savings (70) and reductions in hospital-acquired influenza (71), respectively. Finally, use of the Cepheid Xpert Flu A/B/RSV XC assay in outpatient, clinic-based, physician laboratories improved antiviral utilization (72). These studies support the clinical utility and cost-effectiveness of timely influenza testing.

**Inpatient settings.** Similarly, observational case-control studies of inpatients demonstrated that positive rapid respiratory virus testing was associated with less antibiotic use in both pediatric (73–75) and adult (76) study populations, as well as increased appropriate antiviral use in pediatric and adult populations, compared to patients with negative test results (75–77). When respiratory virus diagnosis via DFA testing was available within 24 h, significant reductions in the duration of hospitalization and antibiotic therapy, as well as the number of microbiological investigations, were observed (78). A simple calculation taking the cost of hospital days saved and subtracting the cost of offering DFA testing yielded a net savings of 400,000 Hong Kong dollars per year in the pediatric population (78). That study was replicated at a U.S. hospital serving a mixed adult/pediatric patient population, and the results were confirmed (79). Such findings have not been limited to DFA panels and conventional influenza rapid antigen tests. Implementation the Cepheid Xpert Flu A/B/RSV XC test for hospitalized adults was associated with decreased length of stay and reduced laboratory utilization (80). In addition, with the use of a laboratory-developed, 16-member, respiratory virus panel for real-time PCR performed within 24 h, hospitalized pediatric patients with positive panel results received fewer antibiotic prescriptions than did patients with negative test results (81). The decreased use of antibiotics for patients with viral infections is an important antimicrobial stewardship endeavor that decreases the overall antibiotic pressure in an environment (e.g., a hospital), thus decreasing the emergence of antibiotic-resistant bacteria.

### Randomized controlled trials.

While these observational studies suggest that timely respiratory virus testing may be cost-effective, the results of RCTs have been mixed.

**Emergency departments and outpatient clinics.** RCTs investigating rapid viral diagnosis in the ED have been primarily performed in pediatric populations. For example, RCTs using rapid influenza antigen tests or a respiratory virus panel for DFA testing were evaluated in otherwise healthy pediatric patients presenting to the ED in three RCTs (82–84) and one quasi-RCT (85). A meta-analysis showed a significant reduction in the number of chest radiographs but only trends toward reductions in the length of ED stay, blood or urine testing, and ED antibiotic use (86). The meta-analysis concluded that there was insufficient evidence to support the use of routine rapid respiratory virus testing in the pediatric ED, although statistical significance for the major outcome measures might not have been reached due to a lack of power (86).

Similarly, an RCT in a pediatric ED evaluating the availability within 12 to 36 h of results from a 17-member respiratory pathogen panel for real-time PCR testing showed no statistically significant differences in hospital admissions, length of hospital stay, or antibiotic use (87). Furthermore, a prospective, 2-arm, randomized study of point-of-care testing using the cobas Liat Flu A/B test in both the pediatric and adult EDs of an academic medical center demonstrated no significant differences in time to discharge or antibiotic use (88). While formal cost analyses were not performed in those studies, the absence of significant differences between study arms suggests that routine rapid viral testing of otherwise healthy children in the pediatric ED may not provide substantial cost savings.

In contrast, the single RCT of rapid influenza testing in the ED that included children with underlying diseases showed that patients with positive influenza results were significantly less likely to undergo routine blood testing or to receive antibiotic prescriptions than were patients who were not tested, although costs were not evaluated (89). Future trials specifically investigating high-risk pediatric populations, including immunocompromised children and children with underlying chronic respiratory and cardiac conditions, may be required to clearly demonstrate statistically significant outcome measures.

Additional RCTs will also be required to investigate the role of rapid respiratory virus testing in the outpatient pediatric setting. In a cluster RCT performed in French outpatient clinics, pediatricians with access to rapid influenza antigen testing prescribed significantly more antivirals but also utilized more antibiotics and performed more chest radiographs than did pediatricians who did not perform rapid antigen tests (90). While the increased use of antibiotics and ancillary testing was primarily for patients with negative rapid antigen test results, the medical necessity of those interventions was not investigated. In the pediatric outpatient setting, the use of rapid tests may thus increase costs. The single RCT of adults presenting to the ED with acute respiratory illness demonstrated that rapid molecular point-of-care influenza A/B testing did not reduce overall antibiotic use, although it was associated with reduced length of stay and improved influenza detection and antiviral use (91).

**Inpatient settings.** To date, a limited number of RCTs have been performed with hospitalized patients. In a trial of nonimmunosuppressed hospitalized adults with lower respiratory tract infections, the availability within 48 h of results from real-time PCR testing with a 14-member panel did not reduce antibiotic use or costs (92). In fact, PCR testing increased average costs by €318 per patient. In a quasi-RCT in both inpatient and outpatient settings, including adults and teenagers, near-care testing with the BioFire FilmArray system did not reduce the hospital length of stay, although influenza-positive patients received antiviral therapy more rapidly than did patients who received routine laboratory-based testing (93). Additional RCTs enrolling low- and high-risk adult and pediatric inpatients will be needed to further evaluate the utility and cost-effectiveness of respiratory virus testing in this clinical setting.

## CONCLUSIONS

As described here, the data supporting the cost-effectiveness of respiratory virus testing are suggestive but far from conclusive. Additional studies are critically important to inform the decision-making of microbiology and virology laboratory medical directors, clinicians, and hospital administrators as they work together to implement respiratory virus testing algorithms that ensure quality, cost-effective, clinical care of patients with suspected respiratory virus infections. In the future, perhaps clinically validated, sophisticated decision analytics incorporating patient age and key risk factors, patient location, test performance and turnaround time, and real-time respiratory virus prevalence data will be available to physicians at the time of test ordering, to help optimize the clinical utility and cost-effectiveness of respiratory virus testing.

## KEY POINTS

• The determination of whether a patient requires respiratory virus testing involves a clinical interpretation that considers presenting signs and symptoms, the day of illness at presentation, and risk factors (such as the extremes of age or immunocompromise) that may predispose patients to severe respiratory disease.
• The timely availability of epidemiological surveillance data may inform clinical decision-making, as respiratory virus prevalence affects the utility of testing.
• The Centers for Disease Control and Prevention recommend that patients who present with a syndrome consistent with influenza and have a negative rapid antigen test result should either receive a confirmatory RT-PCR test or be treated as if they have influenza.
• The American Academy of Pediatrics does not recommend RSV testing for children presenting with bronchiolitis.
• The cost-effectiveness of syndromic panels for respiratory pathogen detection remains an area of active investigation.
• Observational studies suggest that a rapid turnaround time for respiratory virus testing, particularly for influenza, may be a cost-effective testing strategy.
• Randomized controlled trials evaluating a rapid turnaround time for respiratory virus testing in a variety of clinical settings have generated mixed results regarding the clinical utility and cost-effectiveness consistently demonstrated in observational studies.
